# Factors associated with the onset of sexual activity in adolescents: Analytic cross-sectional

**DOI:** 10.15649/cuidarte.3304

**Published:** 2024-05-28

**Authors:** Nancy Milena Sepúlveda Sepúlveda, Diana Isabel Cáceres Rivera, Luis Alberto López Romero, Martha Jeannette Díaz Wandurraga

**Affiliations:** 1 Universidad Cooperativa de Colombia, Bucaramanga, Colombia. nancy.sepulvedas@campusucc.edu.co Universidad Cooperativa de Colombia Universidad Cooperativa de Colombia Bucaramanga Colombia nancy.sepulvedas@campusucc.edu.co; 2 Universidad Cooperativa de Colombia, Bucaramanga, Colombia. dianai.caceres@ucc.edu.co Universidad Cooperativa de Colombia Universidad Cooperativa de Colombia Bucaramanga Colombia dianai.caceres@ucc.edu.co; 3 Universidad Industrial de Santander, Bucaramanga, Colombia. lalopezr@correo.uis.edu.co Universidad Industrial de Santander Universidad Industrial de Santander Bucaramanga Colombia lalopezr@correo.uis.edu.co; 4 Universidad Cooperativa de Colombia, Bucaramanga, Colombia. martha.diaz@campusucc.edu.co Universidad Cooperativa de Colombia Universidad Cooperativa de Colombia Bucaramanga Colombia martha.diaz@campusucc.edu.co

**Keywords:** Adolescents, Risk Factors, Sexual Activity, Adolescente, Factores de Riesgo, Actividad Sexual, Adolescente, Fatores de Risco, Comportamento Sexual

## Abstract

**Introduction:**

Different physical, social, affective, and sexual changes take place during adolescence, such as the sexual debut.

**Objective:**

To explore the factors associated with the onset of sexual activity in adolescents attending school (12-16 years) in Bucaramanga.

**Materials and Methods:**

This analytical cross-sectional study included 440 adolescents from a public school. An instrument developed by Latin American Center for Perinatology - History of Adolescents was applied, and descriptive and bivariate analyses were conducted using a binomial regression model adjusted by age and sex, with sexual activity onset as the outcome and possible associated factors as exposures.

**Results:**

The prevalence of the onset of sexual activity was 22.27% (95% CI: 18.56-26.46). Factors associated with greater prevalence of sexual activity onset were having a criminal record and partner PR=3.24, (95%CI: 2.60-4.05) and PR=2.00, (95%CI: 1.42-2.82), respectively. Male gender PR=1.19, (95% CI: 0.84 1.67), using tobacco PR=1.23 (95% CI: 0.73-2.06), alcohol consumption PR=1.23, (95% CI: 0.73-2.06), and other psychoactive substance use PR=1.78, (95% CI: 0.99-3.19) were risk factors; meanwhile, socializing with friends was a protective factor PR=0.27, (95% CI: 0.20-0.36).

**Discussion:**

Follow-up by parents and school support decrease the risk of adolescent sexual activity onset. Furthermore, interventions in the family and school settings are important.

**Conclusion:**

There are conditions that could promote the beginning of sexual activity in adolescence such as being male, having a partner, having a criminal record, smoking, and consuming alcohol or other substances could promote adolescent sexual activity onset, while socializing with friends was found to be a protective factor.

## Resumen

**Introducción:**

En la adolescencia se producen cambios físicos, sociales, afectivos, sexuales, entre otros; sobre los cambios sexuales, el debut sexual.

**Objetivo:**

Explorar factores asociados al inicio de actividad sexual en adolescentes escolarizados (12 - 16 años) en Bucaramanga.

**Materiales y métodos:**

Estudio de corte trasversal analítico, incluyó 440 adolescentes de un colegio público. Se aplicó el instrumento Centro Latinoamericano de Perinatología - Historia del adolescente. Se realizó un análisis descriptivo de la información y análisis bivariado, empleando un modelo de regresión binomial ajustadas por edad y sexo empleando como desenlace el inicio de actividad sexual, y como exposiciones los posibles factores asociados.

**Resultados:**

La prevalencia del inicio de actividad sexual fue 22,27% (IC 95%: 18,56-26,46). Tener antecedentes judiciales y tener pareja, estaban asociados a mayor prevalencia de inicio de actividad sexual, RP=3,24, (IC 95%: 2,60-4,05) y RP=2,00 (IC 95%: 1,42-2,82), respectivamente. El ser hombre RP=1,19, (IC 95%: 0,84-1,67), consumir tabaco RP=1,23, (IC 95%: 0,73 2,06), alcohol RP= 1,23, (IC 95%: 0,73-2,06) y otras sustancias RP=1,78, (IC 95%: 0,99-3,19) se comportan como factores de riesgo; relacionarse con amigos fue un factor protector RP=0,27, (IC 95%: 0,20-0,36).

**Discusión:**

El acompañamiento de los padres y el apoyo del colegio, disminuyen el riesgo de inicio de actividad sexual en la adolescencia; intervenciones familiares y en el colegio son importantes.

**Conclusiones:**

Existen condiciones que podrían promover el inicio de actividad sexual en la adolescencia tales como, ser hombre, tener pareja, antecedentes personales judiciales, fumar, consumir alcohol y otras sustancias, mientras que, el relacionarse con amigos es un factor protector.

## Resumo

**Introdução:**

Durante a adolescência ocorrem alterações físicas, sociais, emocionais e sexuais, entre outras; sobre mudanças sexuais, estreia sexual.

**Objetivo:**

Explorar fatores associados ao início da atividade sexual em adolescentes escolares (12 - 16 anos) em Bucaramanga.

**Materiais e métodos:**

Estudo transversal analítico que incluiu 440 adolescentes de uma escola pública. Foi aplicado o instrumento Centro Latino-Americano de Perinatologia - História do adolescente. Foi realizada análise descritiva das informações e análise bivariada, utilizando modelo de regressão binomial ajustado por idade e sexo, tendo como desfecho o início da atividade sexual e como exposições os possíveis fatores associados.

**Resultados:**

A prevalência de início da atividade sexual foi de 22,27% (IC 95%: 18,56-26,46). Ter antecedentes judiciais e ter companheiro estiveram associados a maior prevalência de início da atividade sexual, RP=3,24, (IC 95%: 2,60-4,05) e RP=2,00 (IC 95%: 1,42-2,82), respectivamente. Ser homem RP= 1,19, (IC 95%: 0,84-1,67), consumir tabaco RP=1,23, (IC 95%: 0,73 2,06), álcool RP= 1,23, (IC 95%: 0,73-2,06) e outros substâncias RP=1,78, (IC 95%: 0,99-3,19) comportam-se como fatores de risco; interagir com amigos foi fator de proteção RP=0,27; (IC 95%: 0,20-0,36).

**Discussão:**

O acompanhamento parental e o apoio escolar reduzem o risco de início da atividade sexual na adolescência; as intervenções familiares e escolares são importantes.

**Conclusões:**

Existem condições que podem favorecer o início da atividade sexual na adolescência como ser homem, ter companheiro, antecedentes criminais pessoais, fumar, consumir álcool e outras substâncias, sendo o relacionamento com amigos um fator de proteção.

## Introduction

Adolescence is defined as a stage of growth and development during the ages of 10 and 19 years, in between childhood and adulthood^1^. Furthermore, it is defined as the second decade of life^2^^,^^3^ and considered one of the most important transition stages of life, characterized by a rapid pace of growth and increasing maturity^1^. It is also accompanied by rapid changes such as physical^4^, spiritual, and abstract cognitive developments; emotional autonomy from parent figures; development of a new identity; and the onset of the reproductive capacity^2^.

The global adolescent population (aged 15-24 years) is 1.2 billion, the highest in history; of whom 90% live in middle- and low-income countries^3^. Worldwide, the number of adolescents aged 10-19 years is 1.3 billion, with the highest proportion being from India with 21.23%, followed by China with 13.59%, and Latin America and the Caribbean, with 8.67% each^5^.

Human sexuality is a dimension of the personality that is present since conception. Hence, considering its biopsychosocial and spiritual aspects is crucial^6^; in addition, sexuality is an integral part of human life, and everyone manifests and lives it out in different ways^7^. Likewise, sexuality in adolescence is a prioritized aspect of health, where exploration plays a crucial role in physical and emotional development^8^. However, it is normal to experiment in adolescence; therefore, the interest in sexuality increases, especially in love relationships^9^. This is why sexuality and the onset of sexual activity are subjects of interest for research in academia and among the general public^10^.

The onset of sexual activity or sexual debut generally occurs in adolescence, increasing exposure to sexually transmitted diseases (STDs) and unplanned pregnancies^11^. Every year, 357 million new cases of STDs are registered in people aged 15-49 years, with its prevalence varying depending on sex and region. The adolescent fertility rate worldwide is 51 births per 1000 women from 15 to 19 years. The rate in Latin America and the Caribbean is above the world’s average, with the second-highest fertility rate (67 births/1000 adolescents) following Sub-Saharan Africa^12^^,^^15^. However, Colombia has one of the lowest rates, with 57.7 births per 1000 women^12^.

The age of onset of sexual activity is reported differently in function ofthe culture. Some Asian countries have observed a trend in sexual onset after 18 years^16^. A study conducted in the United States found that the age at sexual onset differed according to the culture, revealing that Asians and Caucasians delayed onset, compared to African Americans and Hispanics, whose average reference age was 17 years^17^. In Colombia, the age of highest prevalence ranges between 14 and 16 years old^18^^,^^20^.

Adolescence has been long considered a stage of turmoil and stress, but it is currently regarded as a phase of special opportunities, primarily in terms of positive autonomy^4^. Therefore, the need to reduce sexual risk factors cannot be denied, considering the increasing prevalence of sexual onset during this stage^11^.

Factors associated with the onset ofsexual activity in adolescence include aspects such as culture, family type, and socioeconomic level. Moreover, parents are a source for support for decision-making on this subject, as some factors predispose the onset of sexual activity, such as belonging to reconstructed families rather than nuclear as well as single-parent and extended families^21^^,^^22^. In other cases including some forms of violence, parents or older siblings may force adolescents to obtain money, food, or household items^23^. Earlier onset of sexual activity has also been identified for the following cases, namely, adolescents with a premature first menstrual period and low educational and/or socioeconomic level, drugs and alcohol users, and with parents with a permissive attitude and who received sex education^24^.

Considering the aforementioned findings, researchers and international organizations have expressed their concern about the care of adolescents, especially with respect to sexuality, due to the health implications in the short, medium, and long term. The Adolescent Informatics System was proposed by the Pan-American Health Organization (PAHO) and the World Health Organization (WHO) to assess aspects of sexuality using the Latin American Perinatology Center (CLAP) -Adolescent History instrument, with this intended to improve the quality ofassistance and tackle the comprehensive health challenges of adolescents between 10 and 20 years of age^2^.

Analyzing the factors associated with the onset ofsexual activity in adolescence from a local perspective is necessary, especially with respect to the development of nursing interventions that are adjusted to the context in which the research was developed. Therefore, this study explores factors associated with the onset of sexual activity in adolescents in an educational institution in Bucaramanga, Colombia, as a first step to creating interventions in adolescent health.

## Material and Methods

Design and population: An analytical cross-sectional study was conducted, including 440 adolescents (9 adolescents did not report sexual activity data) at an early and medium stage of secondary education from a public school in the city of Bucaramanga, Colombia, in 2018.

Instrument and measurements: The instrument CLAP-PAHO/WHO-History of Adolescents was employed for data collection^2^. This instrument is divided into different sections, namely, personal background; family background; family, including adolescent siblings, the educational qualification and occupation of the parents, and family perception of the adolescent; housing; education; work and social life; habits; gynecological-urological conditions; and sexuality and psychoemotional situation (see the CLAP-PAHO/WHO-History of Adolescents in Supplementary material). This process was led by two nurses who were trained beforehand and conducted between February and June of 2018.

Data collection process: After the researchers explained about the instrument, the students completed and submitted the printed instrument anonymously. Each participant devoted approximately 1 hour to complete the form.

Statistical analysis: A descriptive analysis of the sociodemographic variables including personal and family background, family variables, education, work, social life, habits, gynecological-urological condition, sexuality, and psychoemotional situation was performed. Subsequently, participants were divided into two groups, namely, those who were sexually active and those who were not. Continuous variables were described as means, and the first and third quartiles were included, as they did not present a normal distribution, except for age and age of menarche/spermarche, according to the Shapiro-Wilk, skewness, and kurtosis tests. The polytomous nominal variables were described as absolute and relative frequencies, including 95% CIs.

Bivariate analysis was conducted to compare adolescents who had begun sexual activity versus those who had not using the chi-square test for categorical variables and Student’s t test for continuous variables. Using simple or crude binomial regression models and providing adjustment by age and sex, possible factors described in the literature as associated with the onset of sexual activity in adolescents were explored. Furthermore, the reasons for prevalence and 95% confidence intervals (CI) were calculated, and p-values below 0.05 were considered significant. Two-tailed statistical tests were performed, and the data were analyzed in the STATA statistical program, version 14.0^25^. Subsequently, it was stored in the PAPYRUS^26^^)^ data repository.

Ethical aspects: This study complied with national and international regulations for research in human beings; adolescents and parents provided informed consent. The research was conducted according to Resolution No. 08430 of 1993 of the Ministry of Health of Colombia, and it was considered “less than minimal risk,” receiving approval from the Ethics Committee of the Universidad Cooperativa de Colombia under act number ID2232.

## Results

An analysis of the variables included in the instrument was performed on 431 male and female adolescents. Nine instruments did not provide any information on the variable onset of sexual activity. The average age of the participants was 14 years; 22.27% (n = 96) (95% CI: 18.56-26.46) of the population of adolescents were sexually active, with 24.29% being male (n = 51) and 20.36% being female (n = 45). The population’s average age was 14.64 ± 1.12 years; the majority of respondents (51.94%, n = 228) were female, who were currently attending ninth grade (32.50%, n = 143). Significant statistical differences were found among the adolescents who were sexually active in relation to the average age (p < 0.001), having a criminal record (p = 0.002), the educational qualification of the father (p = 0.010), using tobacco (p = 0.033), using other psychoactive substances (p = 0.004), and having a partner (p < 0.001). Table 1 presents the sociodemographic characteristics according to the initiation/non-initiation of sexual activity.

**Table 1:** Sociodemographic characteristics of adolescents aged 12-16 years: A cross-sectional approach

Characteristic	All (n = 431)	No sexual activity (n = 335)	Sexually active (n = 96)	p-value*
Age (mean ± SD)	14.64± 1.12	14.49± 1.13	15.15± 0.93	<0.001
Sex				0.328
Female	51.94 (228)	52.54 (176)	46.88 (45)	
Male	48.06 (211)	47.46 (159)	53.13 (51)	
Current school grade				0.001
Sixth	0.45 (2)	0.60 (2)	0.00 (0)	
Seventh	2.50 (11)	2.69 (9)	2.08 (2)	
Eighth	24.32 (107)	27.76 (93)	12.50 (12)	
Ninth	32.50 (143)	34.63 (116)	26.04 (25)	
Tenth	24.09 (106)	20.30 (68)	35.42 (34)	
Eleventh	16.14 (71)	14.03 (47)	23.96 (23)	
Personal background				
Abuse	6.62 (29)	6.01 (20)	9.38 (9)	0.247
Criminal records	1.14 (5)	0.30 (1)	4.21 (4)	0.002
Family background (psychological disorders)				0.145
Yes	10.09 (44)	8.76 (29)	15.63 (15)	
No	88.53 (386)	89.73 (297)	83.33 (80)	
Does not know	1.38 (6)	1.51 (5)	1.04 (1)	
Alcohol/drugs	35.16 (154)	34.43 (115)	41.05 (39)	0.235
Domestic violence				0.614
Yes	8.33 (36)	7.90 (26)	10.64 (10)	
No	91.44 (395)	91.79 (302)	89.36 (84)	
Does not know	0.23 (1)	0.30 (1)	0.00 (0)	
Teenage mother				0.512
Yes	17.51 (76)	18.02 (60)	14.13 (13)	
No	79.26 (344)	78.38 (261)	83.70 (77)	
Does not know	3.23 (14)	3.60 (12)	2.17 (2)	
Family				
Educational qualification of the father or guardian				0.010
None	0.51 (2)	0.00 (0)	2.38 (2)	
Incomplete primary school	2.56 (10)	3.02 (9)	0.00 (0)	
Primary school	24.04 (94)	22.15 (66)	32.14 (27)	
Secondary/technical school	60.87 (238)	61.74 (184)	55.95 (47)	
University	12.02 (47)	13.09 (39)	9.52 (8)	
Educational qualification of the mother or guardian				0.050
Illiterate	0.23 (1)	0.00 (0)	1.10 (1)	
Incomplete primary school	2.11 (9)	2.76 (9)	0.00 (0)	
Primary school	17.61 (75)	15.95 (52)	24.18 (22)	
Secondary/technical school	64.08 (273)	64.42 (210)	61.54 (56)	
University	15.96 (68)	16.87 (55)	13.19 (12)	
Use of psychoactive substances				
Tobacco	2.27 (10)	1.49 (5)	5.21 (5)	0.033
Alcohol	7.05 (31)	5.67 (19)	11.46 (11)	0.500
Other psychoactive substances	5.69 (14)	3.65 (7)	14.29 (7)	0.004
Sexuality				
Need for information	43.49 (187)	40.85 (134)	50.54 (47)	0.096
Age at menarche/spermarche (mean ± SD)	12.26± 1.28	12.26± 1.26	12.20± 1.32	0.837
Use of birth control				0.763
Always	64.29 (45)	100.00 (1)	64.71 (44)	
Sometimes	18.57 (13)	0.00 (0)	17.65 (12)	
Never	17.14 (12)	0.00 (0)	17.65 (12)	
Use of condom				0.827
Always	72.73 (56)	100.00 (1)	72.37 (55)	
Sometimes	22.08 (17)	0.00 (0)	22.37 (17)	
Never	5.19 (4)	0.00 (0)	5.26 (4)	
Social life (acceptance)				0.590
Accepted	95.67 (420)	96.11 (321)	93.75 (90)	
Ignored	1.14 (5)	1.20 (4)	1.04 (1)	
Rejected	1.59 (7)	1.50 (5)	2.08 (2)	
Does not know	1.59 (7)	1.20 (4)	3.13 (3)	
Has a partner	23.09 (100)	18.37 (61)	42.39 (39)	<0.001
Has friends	98.11 (415)	98.75 (316)	96.84 (92)	0.205
Group activity	81.94 (354)	80.79 (265)	84.21 (80)	0.449
Family perception of the adolescent Good	80.92 (352)	82.12 (271)	75.00 (72)	0.249
Regular	17.93 (78)	16.97 (56)	22.92 (22)	
Poor	1.15 (5)	0.91 (3)	2.08 (2)	

*Student’s t test was used for continuous variables, and Pearson’s chi-square test was used for categorical variables. Note: Table 1 was prepared according to the instrument CLAP-PAHO/WHO-History of Adolescents.

Table 2 presents that the variables of personal criminal records, having a partner, and having friends were significantly associated with the onset of sexual activity. Likewise, other possible factors can be observed which, although not significant, demonstrated a possible association, for example, the fact of being a man and the consumption of other psychoactive substances.

**Table 2:** Crude and adjusted values of the factors associated with the onset of sexual activity in adolescents aged 12-16 years

Characteristics	CPR	(95%CI)	P ¥	APR	(95% CI)	P ¥¥
Age	1.60	(1.30-1.867)	<0.001	-	-	-
Sex						
Female	1					
Male	1.19	(0.84-1.67)	0.329	-	-	-
Personal criminal records	3.72	(2.31-5.97)	<0.001	3.24	(2.60-4.05)	<0.001
Teenage mother						
No	1					
Yes	0.77	(0.51-1.16)	0.219	0.80	(0.47-1.35)	0.405
Does not know	0.65	(0.35-1.22)	0.179	0.97	(0.26-3.59)	0.966
Educational qualification of the father or guardian						
Completed primary school or illiterate	1					
Secondary school	0.73	(0.49-1.08)	0123	0.74	(0.51-1.08)	0.121
University	0.61	(0.30-1.23)	0.169	0.57	(0.29-1.14)	0.111
Educational qualification of the mother or guardian						
Completed primary school or illiterate	1					
Secondary school	0.76	(0.51-1.17)	0.219	0.80	(0.47-1.35)	0.405
University	0.65	(0.35-1.21)	0.179	0.97	(0.26-3.58)	0.966
Use of tobacco	1.73	(1.04-2.87)	0.034	1.23	(0.73-2.06)	0.440
Consumption of alcohol	1.73	(1.04-2.87)	0.034	1.23	(0.73-2.06)	0.440
Consumption of other psychoactive substances	2.70	(1.50-4.88)	0.001	1.78	(0.99-3.19)	0.054
Need for information	0.74	(0.52-1.05)	0.096	0.85	(0.60-1.21)	0.368
Acceptance	1.44	(0.73-2.86)	0.296	1.44	(0.77-2.69)	0.256
Has a partner	2.38	(1.68-3.37)	<0.001	2.00	(1.42-2.82)	<0.001
Has friends	0.53	(0.22-1.26)	0.150	0.27	(0.20-0.36)	<0.001
Group activity Family perception of the adolescent	1.21	(0.74-1.98)	0.458	1.19	(0.73-1.94)	0.482
Poor-regular perception	1			1		
Good perception	0.73	(0.49-1.08)	0.112	0.79	(0.54-1.14)	0.208

¥: Value of the simple binomial regression model, ¥¥: Value of the binomial regression model adjusted per age and sex, CPR: crude prevalence ratio, and APR: adjusted prevalence ratio for age and sex.

## Discussion

The study found that a significant percentage of the sample were sexually active adolescents who were attending school. At this stage, between 10 and 19 years old, international recommendations call for such activities to be delayed. The findings revealed that those who had initiated sexual activity tended to have a partner or a criminal record. Males were at greater risk of early sexual activity than females. Likewise, although the statistical findings were not significant, the use of tobacco, alcohol, and other substances tend to be risk factors; by contrast, having friends seems to be a protective factor.

Studies conducted in Latin America and the Caribbean have shown that early onset of sexual activity in adolescents produces a noticeable impact on public health due to early exposure to STDs, unplanned pregnancy, and unsafe abortion^27^^,^^28^, as these are still public health problems given its implications in what forms a hitherto unresolved need^13^^,^^29^. In this context, the school environment is a significant locus for decision-making^21^^,^^30^, where policies have been unable to delay the initiation of sexual activity so far^31^. This implies the need to develop comprehensive sexual education as a fundamental strategy contributing to healthy behavior^32^, even as a friendly strategy with emphasis on Sexual and Reproductive Health Rights (SRHR)^33^^)^ to reduce fears and recognize adolescents as human beings with particular needs.

The most relevant data for this study were related to risk factors of adolescents, such as social relations, where having a partner was identified as a risk for the onset of sexual activity, while having friends was identified as a protective factor. Thus, social weaknesses in socialization are associated with the onset of sexual activity and certain risky sexual behaviors^33^^,^^35^. In this population, follow-up by parents and increased connections with school decrease the risk of the initiation of sexual activity during adolescence, confirming the importance of interventions by families and educational institutions to prevent risky sexual behavior, as described in a study conducted in Ethiopia, where 4 out of every 5 instances of the onset of sexual activity happened at an early stage in an adolescent population with dysfunctional homes^36^.

A noteworthy finding was the greater risk of onset of sexual activity in males (PR: 1.19; CI: 0.84-1.67) than in females, with the former showing a higher prevalence (24.29% in men versus 20.36% ofwomen). A wide-ranging study conducted in India found the same result, where compared with females, twice as many males had had their sexual activity onset. In that study, a greater risk was identified in men who had been exposed to pornography, which may be a common situation in low-income countries^37^.

While the use of tobacco may not have reached a significant value, it showed a trend toward becoming a risk factor. Accordingly, the use of psychoactive substances, such as tobacco, alcohol, and illicit drugs, have been found to develop into a vicious circle, as the use of these substances leads to the initiation of sexual activity. This component exhibits a notable implication for adolescent health, as all of its repercussions for physical and mental health have been widely described, including anxiety, obesity, and depression, in addition to suicide attempts^38^^,^^40^.

Finally, this study had a limitation related to the attitudes of the adolescent participants, who were shy when it comes to talking about sexuality; this observation could be due to the lack of spaces for open dialogue concerning sexuality at schools and colleges. While suggestions have been made regarding the approach and contents that should be comprehensively addressed^41^, the need for schools and families to take an active role in education and dialogue must be emphasized. The aim is to make a difference in adolescents’ sexual health, which is simultaneously related to other prevailing phenomena at schools that are already being addressed, such as bullying and absenteeism, which play a role in the onset of sexual activity^42^.

Among the main strengths of this study are the size of the sample and the use of the instrument proposed by the PAHO/WHO, which support data collection of a large number of variables that can explain other risk factors in the adolescent population under study. Using these data, interventions can be planned that can improve the health of a target group of adolescents. However, due to the cross sectional approach of this study, its results cannot be used to establish causality in the analysis; likewise, as the data were collected through a self-report survey, bias in the measurements cannot be excluded. Future prospective studies should be carried out to overcome these limitations.

## Conclusions

This study revealed the onset of sexual activity in adolescence. Having a criminal record and having a partner were identified as risk factors during data collection; meanwhile, socializing with friends was identified as a protective factor for early sexual debut. These findings allow us to propose interventions directed to the specific needs of the adolescent population and to the context in which they develop.
